# Supplementary material for: Interspecific and Environmental Influence on the Foliar Metabolomes of Mitragyna Species Through Recursive OPLSDA Modeling
Source: Plants (Basel). 2025 Sep 1;14(17):2721. doi: 10.3390/plants14172721 (PMC12430465; doi:10.3390/plants14172721)
Supplement: Supplementary file 1 [file plants-14-02721-s001.zip › Doc 1.pdf]

Model testing with IRIS dataset.

The classification performance of this framework was first tested on the Iris dataset [1]. This dataset is widely used as a benchmark in statistics and machine learning studies related to multi-class characterization. It consists of three classes of different iris plant species that include *Setosa*, *Versicolor*, and *Virginica*. While one class is distinctly separable (*Setosa*), the other two exhibit overlapping features, making them a challenge for classification.

The effectiveness in separation of multiple classes was modeled through of hierarchical OPLS-DA was first tested using the Iris dataset. The Iris dataset contains 50 samples each of three species of the Iris flower; *Setosa*, *Versicolor*, and *Virginica*, with four morphological features: sepal length, sepal width, petal length, and petal width, used to distinguish between the species. *Setosa* is linearly separable from the other two classes, while *Versicolor* and *Virginica* exhibit some overlap, making the dataset ideal for evaluating classification models. Figure S2 illustrates the OPLS-DA score plots for the two sequential splits predicted by the hierarchical classification model, indicative of a progressive refinement in class identification.

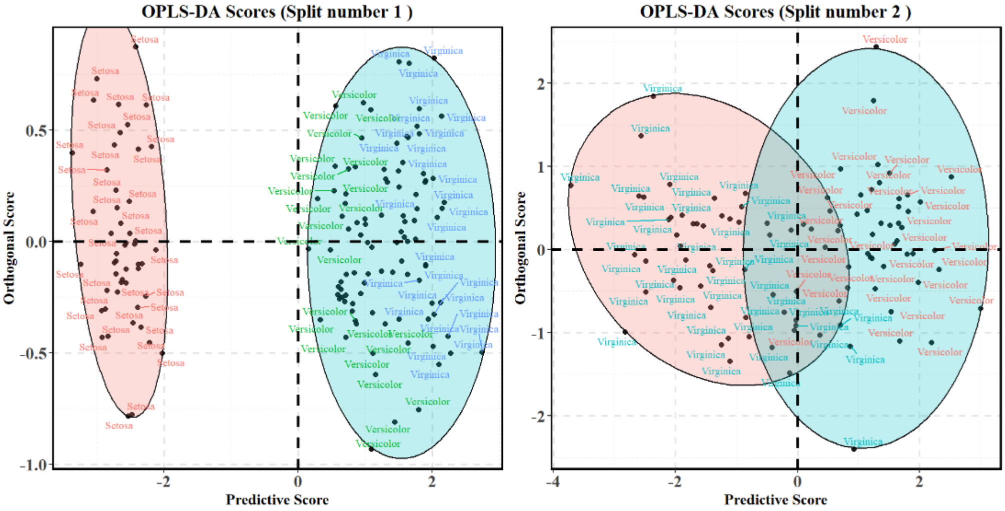

Figure S2 OPLS-DA score plots for two recursive splits in the hierarchical classification framework. The first split (left) led to a complete separation of the *Setosa* class from the other two classes (combined *Versicolor* and *Virginica* classes). The second split (right) attempted to differentiate *Versicolor* from *Virginica*, with partial overlap obtained between the two classes, suggesting some species similarity as measured by the traits. Ellipses represent the 95% confidence regions for each class.

At the first split, the *Setosa* class was entirely separated from the remaining samples consisting of *Versicolor* and *Virginica* samples. A clear separation of the *Setosa* from the other classes was indicated by the robustness of the split parameters (as quantified by the OPLSDA *p-value* < 0.05) for split 1 and 2, respectively. In the second split, the OPLS-DA model attempts to distinguish between Versicolor and *Virginica*, leading to a more complex separation pattern. Unlike the first split, a noticeable overlap was observed between the two classes in split 2, which contained *Versicolor* and *Virginica*, indicative of species similarity.

References

1. Fisher, R.A. The Use of Multiple Measurements in Taxonomic Problems. *Ann. Eugen.* **1936**, *7*, 179–188.
