# Supplementary material for: Interspecific and Environmental Influence on the Foliar Metabolomes of Mitragyna Species Through Recursive OPLSDA Modeling
Source: Plants (Basel). 2025 Sep 1;14(17):2721. doi: 10.3390/plants14172721 (PMC12430465; doi:10.3390/plants14172721)
Supplement: Supplementary file 1 [file plants-14-02721-s001.zip › Figure S1.pdf]

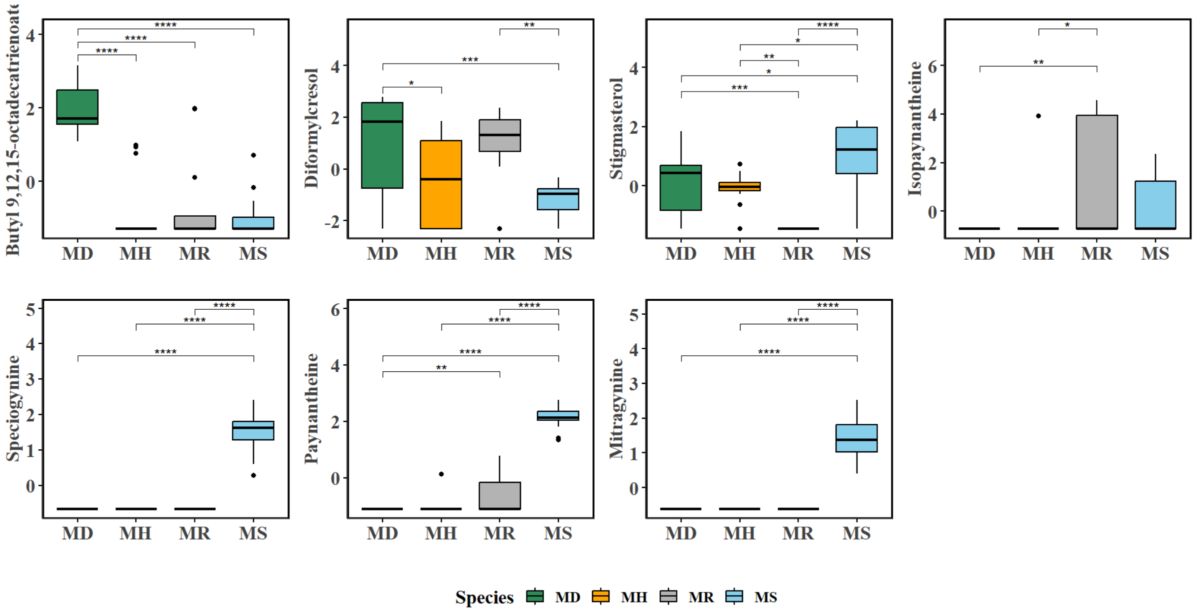

Figure S1. Boxplots of metabolite presence that significantly loaded the principle axes in the PCA plot. The bar plots include normalized values (mean +/- one Standard Deviation), with the boxes ranging from the 25% and the 75% percentiles, 5% and 95% percentiles indicated as error bars, and horizontal lines within boxes indicating median values, while the dots indicate outliers. The asterisks in various subplots indicate statistically significant difference in the qualitative presence of the given metabolite between the four species. The four *Mitragyna* species are indicated by abbreviations as MD: *M. diversifolia*, MH: *M. hirsuta*, MR: *M. rotundifolia*, and MS: *M. speciosa*.
