# Supplementary material for: Interspecific and Environmental Influence on the Foliar Metabolomes of Mitragyna Species Through Recursive OPLSDA Modeling
Source: Plants (Basel). 2025 Sep 1;14(17):2721. doi: 10.3390/plants14172721 (PMC12430465; doi:10.3390/plants14172721)
Supplement: Supplementary file 1 [file plants-14-02721-s001.zip › Table S2.pdf]

Table S2. Metrics for pairwise OPLSDA of the *Mitragyna* foliar metabolome. The models with significant *p-value* are highlighted in green.

Class 1	Class 2	OPLS-DA *p-value*	Comments	R^2 (Q^2)
MD North	MD South	0.01	Significant	0.84 (0.41)
	MH North	0.34	Not Significant	NA
	MH South	0.37	Not Significant	NA
	MR North	0.99	Not Significant	NA
	MR South	0.16	Not Significant	NA
	MS North	0.55	Not Significant	NA
	MS South	0.54	Not Significant	NA
MD South	MD North	-	-	-
	MH North	0.25	Not Significant	NA
	MH South	0.43	Not Significant	NA
	MR North	0.94	Not Significant	NA
	MR South	0.33	Not Significant	NA
	MS North	0.91	Not Significant	NA
	MS South	0.89	Not Significant	NA
MH North	MD North	-	-	-
	MD South	-	-	-
	MH South	0.07	Not Significant	NA
	MR North	0.80	Not Significant	NA
	MR South	0.33	Not Significant	NA
	MS North	0.80	Not Significant	NA

	MS South	0.91	Not Significant	NA
MH South	MD North	-	-	-
	MD South	-	-	-
	MH North	-	-	-
	MR North	0.04	Significant	0.99 (0.88)
	MR South	0.08	Not Significant	NA
	MS North	0.86	Not Significant	NA
	MS South	0.76	Not Significant	NA
MR North	MD North	-	-	-
	MD South	-	-	-
	MH North	-	-	-
	MH South	-	-	-
	MR South	0.39	Not Significant	NA
	MS North	0.18	Not Significant	NA
	MS South	0.68	Not Significant	NA
MR South	MD North	-	-	-
	MD South	-	-	-
	MH North	-	-	-
	MH South	-	-	-
	MR North	-	-	-
	MS North	0.88	Not Significant	NA
	MS South	0.93	Not Significant	NA
	MD North	-	-	-

MS North	MD South	-	-	-
	MH North	-	-	-
	MH South	-	-	-
	MR North	-	-	-
	MR South	-	-	-
	MS South	0.37	Not Significant	NA
