# Supplementary material for: Interspecific and Environmental Influence on the Foliar Metabolomes of Mitragyna Species Through Recursive OPLSDA Modeling
Source: Plants (Basel). 2025 Sep 1;14(17):2721. doi: 10.3390/plants14172721 (PMC12430465; doi:10.3390/plants14172721)
Supplement: Supplementary file 1 [file plants-14-02721-s001.zip › Table S3.pdf]

Table S3. Cohen's d effect sizes for some metabolites associated with significant discrimination at each hierarchical split in the OPLS-DA model as well as reported previously in literature. The table lists metabolites with corresponding magnitude of effect size (|d|), as well as categorization (negligible, small, medium, large) for the four significant splits as obtained by the hierarchal clustering. The interpretation of estimates are based on absolute thresholds of negligible (d < 0.2), small (d > 0.2), medium (d > 0.5), or large (d > 0.8). Large effect sizes (d > 0.8) indicate substantial differences in metabolite abundance between clusters at a given split.

**Hierarchal Split number**	**Metabolite name**	**Absolute d estimate**	**Magnitude of effect size**
1	mitragynine	2.159362	Large
	paynantheine	3.281626	Large
	speciociliatine	2.89233	Large
	isopaynantheine	0.694596	Medium
	mitraphylline	0.8166463	Large
	isorhynchophylline	1.024687	Large
	5-hydroxymethylfurfural	0.9633254	Large
2	Benzoic acid, 2,3-dihydroxy-, methyl ester	4.572821	Large
	Catechol	2.096281	Large
	hexamethyl-cyclotrisiloxane	5.128028	Large
	Eugenol	0.9756247	Large
	Methyl salicylate	3.448052	Large

	Vanillic acid	1.307366	Large
	Vitamin E	0.6691926	Medium
3	isorhynchophylline	1.057896	Large
	eugenol	1.062883	Large
	octadecanoic acid	0.5849205	Medium
	stigmasterol	0.4429498	Small
	mitraphylline	0.7621079	Medium
	phenol derivatives	1.287383	Large
	squalene	0.305283	Small
4	Ajmalicine	1.812697	Large
	2,5-Dimethylfuran-3,4(2H,5H)-dione	0.8289876	Large
	3-methyl-2,5-Furandione	0.9772013	Large
	2-Furancarboxylic acid	0.5913286	Medium
	2,5-diethoxytetrahydro-Furan	1.849322	Large
	Furaneol	1.084988	Large
	Catechol	1.074894	Large
	D-Allose	1.08241	Large

	Megastigmatrienone	1.714046	Large
	Thymine	1.380491	Large
